# Effects of Regular Kefir Consumption on Gut Microbiota in Patients with Metabolic Syndrome: A Parallel-Group, Randomized, Controlled Study

**DOI:** 10.3390/nu11092089

**Published:** 2019-09-04

**Authors:** Ezgi BELLIKCI-KOYU, Banu Pınar SARER-YUREKLI, Yakut AKYON, Fadime AYDIN-KOSE, Cem KARAGOZLU, Ahmet Gokhan OZGEN, Annika BRINKMANN, Andreas NITSCHE, Koray ERGUNAY, Engin YILMAZ, Zehra BUYUKTUNCER

**Affiliations:** 1 Department of Nutrition and Dietetics, Faculty of Health Sciences, Hacettepe University, Ankara 06230, Turkey; 2 Department of Nutrition and Dietetics, Faculty of Health Sciences, Izmir Katip Celebi University, Izmir 35620, Turkey; 3 Department of Endocrinology, Faculty of Medicine, Ege University, Izmir 35040, Turkey; 4 Department of Medical Microbiology, Faculty of Medicine, Hacettepe University, Ankara 06230, Turkey; 5 Department of Biochemistry, Faculty of Pharmacy, Ege University, Izmir 35040, Turkey; 6 Department of Dairy Technology, Faculty of Agriculture, Ege University, Izmir 35040, Turkey; 7 Robert Koch Institute; Center for Biological Threats and Special Pathogens 1 (ZBS-1), Berlin 13353, Germany; 8 Department of Medical Biology, Acıbadem Mehmet Ali Aydınlar University, Istanbul 34752, Turkey

**Keywords:** kefir, gut microbiota, metabolic syndrome

## Abstract

Several health-promoting effects of kefir have been suggested, however, there is limited evidence for its potential effect on gut microbiota in metabolic syndrome This study aimed to investigate the effects of regular kefir consumption on gut microbiota composition, and their relation with the components of metabolic syndrome. In a parallel-group, randomized, controlled clinical trial setting, patients with metabolic syndrome were randomized to receive 180 mL/day kefir (*n* = 12) or unfermented milk (*n* = 10) for 12 weeks. Anthropometrical measurements, blood samples, blood pressure measurements, and fecal samples were taken at the beginning and end of the study. Fasting insulin, HOMA-IR, TNF-α, IFN-γ, and systolic and diastolic blood pressure showed a significant decrease by the intervention of kefir (*p* ≤ 0.05, for each). However, no significant difference was obtained between the kefir and unfermented milk groups (*p* > 0.05 for each). Gut microbiota analysis showed that regular kefir consumption resulted in a significant increase only in the relative abundance of *Actinobacteria* (*p* = 0.023). No significant change in the relative abundance of *Bacteroidetes, Proteobacteria or Verrucomicrobia* by kefir consumption was obtained. Furthermore, the changes in the relative abundance of sub-phylum bacterial populations did not differ significantly between the groups (*p* > 0.05, for each). Kefir supplementation had favorable effects on some of the metabolic syndrome parameters, however, further investigation is needed to understand its effect on gut microbiota composition.

## 1. Introduction

Metabolic syndrome (MetS) is a pathologic condition that includes abdominal obesity, insulin resistance, dyslipidemia, and arterial hypertension [1]. Each component of MetS is known as a risk factor for the development of type 2 diabetes and cardiovascular diseases. It was found that the risk of type 2 diabetes was five times, the risk of cardiovascular disease was two times, and the risk of death was one-half times higher in individuals with MetS compared to those without the syndrome. Due to its high prevalence and related health problems, the MetS is currently considered as a significant public health problem [1,2].

MetS has a multi-factorial etiology comprising complex interactions between genetic predispositions and environmental factors including diet, physical activity, and other lifestyle factors [3,4]. Since Turnbaugh et al. showed the linked between gut microbiota and obesity, there has been growing evidence that suggests a causal relationship between gut microbiota and the components of MetS [5]. Primary, the low-grade chronic inflammation state in MetS has been explained by the metabolic endotoxemia that was a result of gut dysbiosis [6,7]. Most of the animal and human studies have reported that obesity and insulin resistance are associated with an altered ratio of *Firmicutes* and *Bacteroidetes* [8,9]. In addition to the effects on immune function, the gut microbiota also exerts its role through the influence on host energy metabolism and gut barrier integrity [10,11]. Therefore, the gut microbiota has been suggested as a potential target to modify the risk factors that contribute to conditions of MetS. 

The modification of diet using prebiotics and probiotics has been suggested as a useful strategy to improve metabolic health via the modulation of gut microbiota. Although the effects of probiotic and prebiotic supplementation on metabolic health have been examined in previous studies, the results are inconsistent due to the choice of probiotic strain, formulation of the probiotic, outcome of interest, and duration of the intervention [12,13,14]. Furthermore, ingestion of probiotics through traditional fermented foods has not been widely examined in terms of their efficiency on MetS components. Kefir is a fermented milk product, traditionally produced with kefir grains that have a specific combination of bacteria and yeasts [15,16]. Microbial composition of kefir varies depending upon the type of kefir grains, the type and composition of milk, culture medium, fermentation period and temperature, and also storage conditions [17]. *Lactobacillus*, *Lactococcus, Streptococcus Leuconostoc,* and acetic acid bacteria are the most common bacteria; and *Saccharomyces*, *Kluyveromyces,* and *Candida* species are mostly found yeasts in kefir [18]. Animal studies have suggested that kefir has anticarcinogenic, antimicrobial, anti-inflammatory activities, and thus may ameliorate MetS components [19,20,21,22,23]. However, there is still limited clinical evidence for its potential effects on MetS patients. To our knowledge, especially, the effects of kefir on MetS components via the modulation of gut microbiota have not been examined widely in clinical settings. To address the research gap, this study aimed to investigate the effects of daily kefir consumption on gut microbiota composition and their relation with the components of metabolic syndrome in adults with MetS. 

## 2. Materials and Methods 

### 2.1. Subjects

Subjects with MetS, aged 18–65 years, were recruited from the outpatient clinic of the Department of Endocrinology and Metabolism at the Ege University, Izmir, Turkey. MetS was diagnosed using the IDF-2005 guidelines [24]. The eligibility of a subject was confirmed following a physical examination by the research endocrinologist and a nutritional assessment by the research dietitian in the screening period. Adults were excluded if they (1) were using antibiotics in the past 1 month or during the intervention period, (2) were using dietary supplements (probiotic, prebiotic, or symbiotic) during the past three months or during the intervention period, (3) were pregnant or lactating, (4) had severe liver, kidney, heart, or immune deficiency, (5) had chronic gastrointestinal system diseases, type 1 diabetes or cancer, (6) had allergy to the dairy products or lactose intolerance, (7) were currently taking prescribed drugs that can modulate lipid profile or glycaemic control, and (8) did not comply with the consumption of test drinks. 

The compliance was assessed by interviewing the participants and reviewing the record of their consumption in each visit. Non-compliance was defined as consuming < 80% of the scheduled serving during the study period. 

The study protocol was approved by the Ethics Committee of Clinical Research at Ege University Faculty of Medicine (15-2.1/14) and registered at clinicaltrials.gov (NCT03966846). All procedures were performed in accordance with the Declaration of Helsinki. Written informed consent was obtained from all participants.

### 2.2. Study Design 

**Figure 1:**
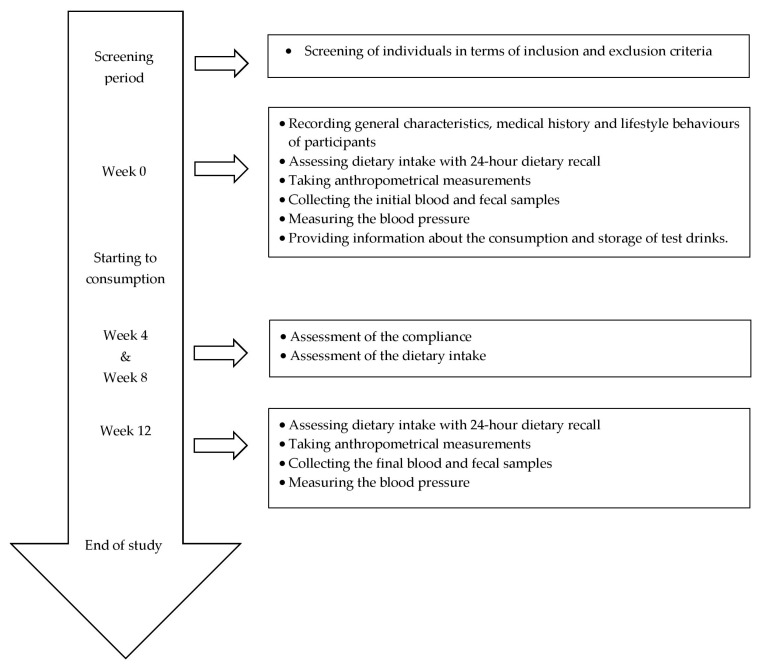
Timeline of the study.

A parallel-group, randomized, controlled clinical trial was performed. A total of 40 eligible participants were randomized, and 20 participants in each group were allocated to intervention. Five participants in the kefir group and four participants in the unfermented milk group did not receive the allocated intervention due to medical conditions and not providing the fecal samples, and also three participants in the kefir group and six participants in the unfermented milk group discontinued the intervention due to taking antibiotics and declined consent. Therefore, the study was completed with 22 participants and an allocation ratio of 55%. The recruitment and follow up of participants were conducted between March 2015–July 2017. Participants were randomized into two groups (kefir group and unfermented milk group as control) by the research physicians using a stratified block randomization method. The random allocation sequence was provided by the Department of Biostatistics, Hacettepe University. Participants visited the research center 5 times in total. The first visit included the screening of individuals in terms of inclusion and exclusion criteria. The second visit (Week 0) included recording general characteristics, medical history, and lifestyle behaviors of participants, assessing the nutritional status of participants using 24-h dietary recall and anthropometrical measurements, collecting the initial blood and fecal samples, measuring the blood pressure, and also proving information about the consumption and storage of test drinks. The third (Week 4) and forth (Week 8) visits included the assessment of the compliance in terms of consumption of test drinks and dietary intake. The fifth visit (Week 12) included the assessment of the nutritional status of participants using 24-h dietary recall and anthropometrical measurements, collection of the final blood and fecal samples, and the measurement of blood pressure (Figure 1). 

The primary outcome of the study was the change in the relative abundance of microorganisms in gut microbiota by regular kefir consumption. The potential correlations between the changes in dietary intake, anthropometrical measurements, biochemical parameters, or blood pressure and the change in microbiota composition were all secondary outcomes. 

### 2.3. Intervention

During a 12-week intervention period, kefir group (*n* = 12) received kefir (180 mL/day) while control group (*n* = 10) received unfermented milk (180 mL/day) regularly. Participants were asked to maintain their habitual diet and physical activity. Additional products that contain probiotics were not allowed during the intervention period. No dietary supplement use was recorded before or during the study. 

### 2.4. Test Drinks

Two dairy products (kefir and unfermented milk) were tested in parallel groups. Kefir was prepared using the culture of DC1500I (Danisco, Olsztzyn, Poland) containing *Lactococcus lactis* subsp. *lactis, Lactococcus lactis* subsp. *cremoris, Lactococcus lactis* subsp. *diacetylactis, Leuconostoc mesenteroides* subsp. *cremoris, Lactobacillus kefir, Kluyveromyces marxianus*, and *Saccharomyces unisporus* at Ege University Faculty of Agriculture, Department of Dairy Technology. Kefir was derived from the full-fat (3.5%) homogenized and pasteurized (at 85 °C) milk that was used as a control drink at the same time. The beverages were distributed and stored at 4 °C. The test drinks were received to participants twice a week, and they consumed the test drinks between 1 and 4 days of post-production. 

### 2.5. Dietary Assessment 

Dietary intake was assessed using 24-h dietary recall method by research dietician in each visit. A photographic atlas of food portion sizes was used to clarify the amounts of food items consumed. Dietary energy, macro- and micronutrient intakes were analyzed using BeBIS software (Ebispro for Windows, Stuttgart, Germany; Turkish Version BeBIS, Nutrition Information System, Version 8).

### 2.6. Anthropometrical Measurements 

Body weight and composition (fat mass and fat-free mass) were measured by Tanita BC418 (USA), and height was measured by a calibrated stadiometer (Nan Tartı, TR). Body Mass Index (BMI) was calculated by dividing body weight (in kilograms) by the square of height (in meter). The waist circumference was measured at the midpoint between the lower ribs and the iliac crest, and hip circumference was measured horizontal at the largest circumference of hip. Waist-to-hip ratio (WHR) was calculated. 

### 2.7. Biochemical Analysis and Blood Pressure 

Venous blood samples were drawn after a 10-h overnight fasting excluding only the water at the Visit 1, Visit 2 (Week 0), and Visit 5 (Week 12). Serum glucose, insulin, HbA1c, total cholesterol, high-density lipoprotein (HDL-C), low-density lipoprotein (LDL-C), triglycerides, homocysteine, high-sensitivity C-reactive protein (hs-CRP), alanine aminotransferase (ALT), aspartate aminotransferase (AST), and gamma-glutamyl transferase (GGT) were analyzed at Ege University, Hospital of Medical School, Laboratory of Clinical Biochemistry. All biomarkers were analyzed using routine methods by Roche/Cobas analyzer series. Serum concentrations of tumor necrosis factor-α (TNF-α), interleukin-6 (IL-6), interleukin-10 (IL-10) and interferon-gamma (IFN-γ) were determined by enzyme-linked immunosorbent assay (ELISA) using standard kits, and the analyses were conducted as described by the manufacturer (DIAsource ImmunoAssays, Louvain-la-Neuve, Belgium). Insulin resistance was assessed using Homeostatic Model Assessment (HOMA-IR) model calculated with the equation of “the fasting insulin level (μU/L) × fasting plasma glucose (mg/dL)/405”. Systolic (SBP) and diastolic blood pressure (DBP) were measured at the brachial artery of right upper arm after 15 min rest. Both blood pressures were measured twice at 5-min intervals and recorded on average.

### 2.8. Specimen Processing, 16S rRNA Amplification and Sequencing 

Fecal samples from individuals enrolled in the study were collected in sterile containers and kept frozen at −80 °C. A sterile spatula was used to obtain 4–5 pieces of frozen chunks from the surface and internal portions of the specimen. They were combined for a 150–200 mg total weight for each and mixed by vortexing. Following a bead-beated step described by Tomas et al. and Wu et al. [25,26], DNA was extracted using Qiagen Stool Mini Kit (Qiagen, Hilden, Germany) as directed by the manufacturer. DNA amount of 50 ng/µL was prepared for each specimen, using a NanoDrop 2000 spectrophotometer (Thermo Fisher Scientific, Hennigsdorf, Germany).

The 16S rRNA sequences were amplified using previously-described primers targeting the V3-V4 region, frequently used to study bacterial diversity [27], with Illumina adapter overhang sequences added, as directed by the manufacturer. Attachment of sequencing adapters to PCR products, amplification and library preparation were performed using the Nextera XT Index and Nextera DNA Library Prep kits (Illumina, San Diego, CA, United States), as suggested by the manufacturer. Product clean-up, library quantification, and optimization were carried out using the Agencourt AMPure XP reagent (Beckman Coulter Biosciences, Krefeld, Germany) standard protocol and a Qubit 2.0 Fluorometer (Thermo Fisher Scientific, Rochester, NY, USA). The sequencing runs were performed in an Illumina MiSeq sequencer (Illumina Inc., New York, USA). 

### 2.9. Data Handling, Phylogenetic and Statistical Analyses

The raw sequencing data were de-multiplexed and extracted in fastq format. Sequence data handling and taxonomic assignment were carried out using Geneious v11.1 (Biomatters Ltd., Auckland, New Zealand), MALT V0.3.8 and MEGAN v6.11 [28]. Trimming for read quality and length and adaptor sequence removal were performed using Trimmomatic v0.35 [29,30]. Trimmed reads were mapped to the NCBI-NT RefSeq 16S database via MALT V0.3.8, with hits down to 95% identity. For the operational taxonomic unit (OTU) identification and taxonomic binning, LCA-assignment algorithm (with 95% minimum identity) and 16s percent identity filter (species assignment at 99% identity) were employed. Relative bacterial abundance on the genus and species levels were calculated using the reads numbers of the corresponding OTUs.

Various alpha and beta diversity metrics were calculated for bacterial diversity and composition analyses. For this purpose, raw data were imported into QIIME2 [31], filtered and controlled for quality and chimeric sequences using DADA2, q2-demux, and dblur scripts [32,33]. The trimmed reads were subsequently mapped to the GreenGenes [34] and SILVA [35] databases for OTU identification and taxonomic binning. Faith phylogenetic diversity (PD), Pielou, Shannon, and Jaccard indices and Bray–Curtis and UniFrac distances were computed and evaluated using Kruskal–Wallis, Spearman, or permutational multivariate analysis of variance (PERMANOVA) tests as appropriate.

Data were analyzed using the Statistical Package for the Social Sciences (SPSS), version 22.0. (SPSS Inc., Chicago, IL, USA). Data normality was tested by Shapiro–Wilk test prior to further analyses. Kruskal–Wallis and Mann–Whitney U tests were employed for comparisons among groups where appropriate. Spearman’s rank-order correlation was used to analyze the correlation analysis between microbial taxa and biochemical and blood pressure measurements. A value of *p* < 0.05 was considered as significant.

## 3. Results

Baseline characteristics of the participants were summarized in Table 1. There were no differences in terms of age, dietary intake, anthropometrical measurements, and biochemical parameters except serum insulin levels between groups.

The changes in dietary intake, anthropometrical measurements, biochemical parameters, and blood pressure during the intervention period were given in Table 2. Intakes of energy and macronutrients did not change significantly during the intervention period in either kefir or unfermented milk groups (*p* > 0.05, for each). In terms of anthropometrical measurements, body weight and fat mass showed slight reductions after the 12-weeks intervention of kefir compared to unfermented milk, however, the changes in any of anthropometrical measurement from baseline to after the intervention did not differ significantly between the groups (*p* > 0.05, for each). Among the biochemical biomarkers, almost all parameters of lipid profile and glycaemic status showed amelioration by the intervention kefir group, however, only the difference in fasting insulin and thereby HOMA-IR from baseline to after intervention was significant (*p* = 0.050). Furthermore, TNF-α and IFN-γ showed a significant decrease after the intervention of kefir (*p* = 0.015 and *p* = 0.013, respectively), whereas IL-6 showed a slightly larger decrease in the unfermented milk group (*p* = 0.047). Both systolic blood pressure and diastolic blood pressure decreased significantly after the intervention in the kefir group (respectively *p* = 0.041 and *p* = 0.019), while only systolic blood pressure showed a modest decrease in the unfermented milk group (*p* = 0.047).

**Figure 2:**
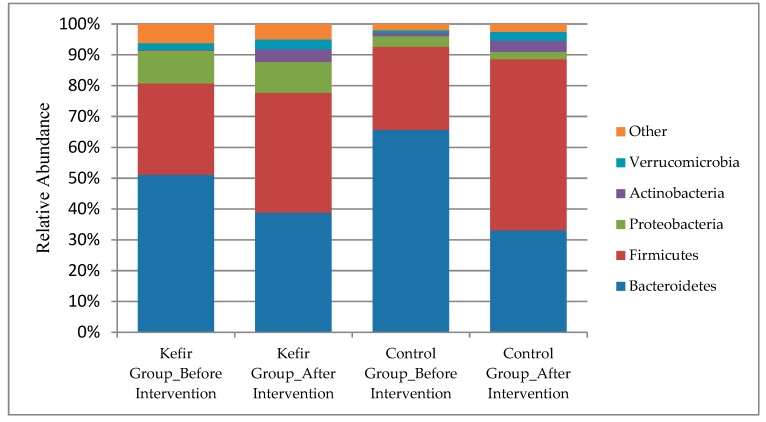
Gut microbiota composition before and after the intervention in each group.

In regard to the analysis of gut microbiota composition, the mean number of total reads per sample was 90627 (Standart Deviation (SD): 44912; range: 31198–183068) at baseline and 118025 (SD:38831; range 39248–171915) after the intervention in kefir group, while it was recorded as 138775 (SD: 29961, range: 100922–195456) at the baseline and 95058 (SD: 23740, range: 55853–124889) after the intervention in the unfermented milk group. The gut microbiome of the participants was composed of phyla *Bacteroidetes* (51%), *Firmicutes* (30%), *Proteobacteria* (11%), *Verrucomicrobia* (0.02%) and *Actinobacteria* (0.003%) at the baseline in kefir group; the relative abundance of these phyla were detected as 39%, 39%, 10%, 0.03%, and 0.04% respectively after the intervention of kefir. Only the increase in the relative abundance of *Actinobacteria* was found to be statistically significant (*p* = 0.023). In the unfermented milk group, the phyla *Bacteroidetes* (66%), *Firmicutes* (27%), *Verrucomicrobia* (0.01%), *Actinobacteria* (0.01%), and *Proteobacteria* (0.03%) were detected at the baseline, however, the relative abundance of these phyla were changed to 33%, 56%, 0.03%, 0.04%, and 0.02% respectively after the intervention (Figure 2). When the changes in the relative abundance of each phyla distribution from after the intervention to baseline were compared between the groups, no significant difference was obtained (*p* > 0.05, for each). In the kefir group, the median *of Firmicutes/Bacteroidetes* ratio was 0.62 (range:0.06–10.01) at the baseline, and 1.77 (range:0.14–46.16) after the intervention (*p* = 0.388). This ratio was 0.30 (range:0.03–25.27) and 2.22 (range:0.34–12.04) respectively at baseline and end of the intervention in the unfermented milk group (*p* = 0.333). No significant difference was obtained between the changes in *Firmicutes* to *Bacteroidetes* ratio of groups (*p* > 0.05).

**Figure 3:**
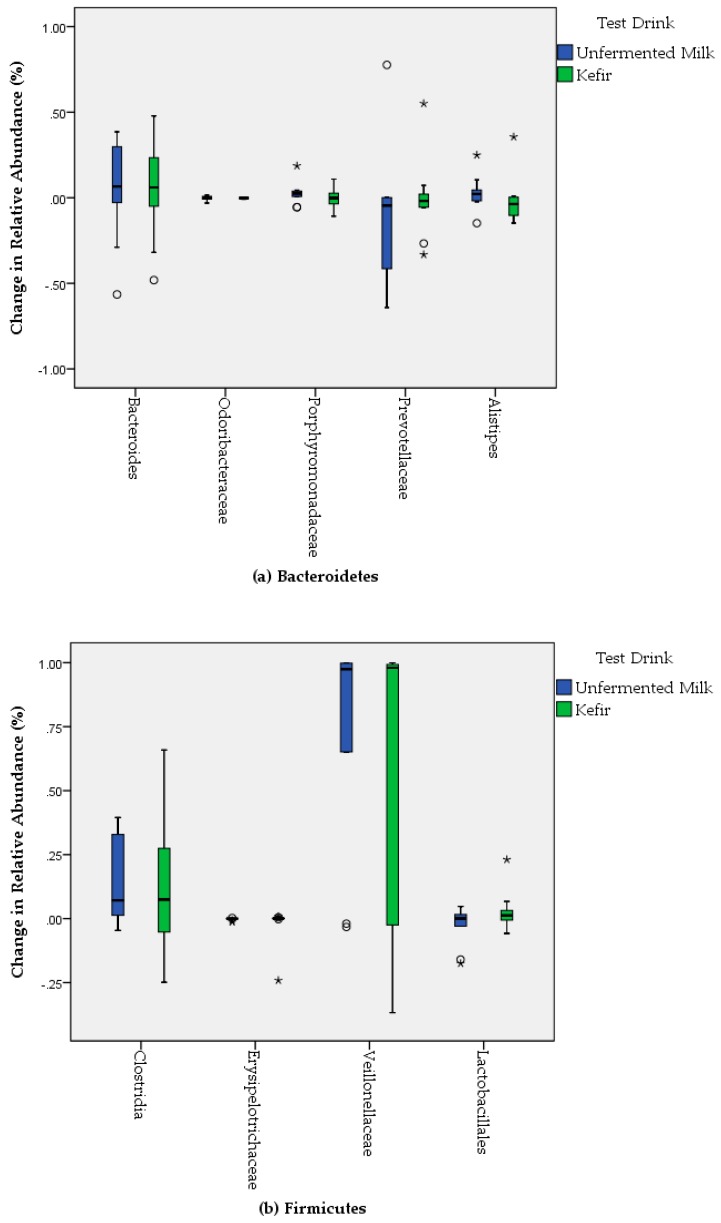
Bacterial changes in the relative abundance of Bacteriodetes (**a**) and Firmucutes (**b**) by the consumption of test drinks.

The changes in the relative abundance of *Bacteriodetes* and *Firmucutes* by the consumption of test drinks were given in Figure 3. The phyla *Bacteroidetes* was composed of five dominant genera; *Bacteroides*, *Odoribacteraceae*, *Porphyromonadaceae*, *Prevotellaceae*, and *Alistipes* in the gut microbiome of participants. *Bacteroides* (54% in kefir group and 44% in unfermented milk group), *Prevotellacea* (26% in kefir group and 40% in unfermented milk group) and *Alistipes* (11% in kefir group and 7% in unfermented milk group) were respectively most abundant families among *Bacteroidetes* at baseline. The relative abundance of *Bacteroides* changed to 59% in kefir group and 50% in unfermented milk group; *Prevotellacea* changed to 25% in kefir group and 29% in unfermented milk group; *Alistipes* changed to 9% in kefir group and 10% in unfermented milk group after the intervention. Despite the modest changes in the relative abundance of some genera, no significant difference was obtained when the changes in the relative abundances were compared between groups (*p* > 0.05, for each). Among *Firmicutes*, *Clostridia*, *Erysipelotrichaceae*, *Veillonellaceae,* and *Lactobacillales* were obtained in the fecal samples of participants. Although an increase in the relative abundance of *Clostridia* (from 73% to 85%) and *Lactobacillales* (2% to 5%), and also a decrease in the relative abundance of *Veillonellaceae* (from 9% to 6%) were obtained from baseline to after the intervention in kefir group, none of these changes were statistically significant (*p* > 0.05, for each). In the unfermented milk group, the relative abundance of *Clostridia* was increased from 75% to 89%, whereas *Lactobacillales* (5% to 2%) and *Veillonellaceae* (from 5% to 4%) were decreased from baseline to after the intervention. Similar to the phyla *Bacteriodetes*, no significant difference was obtained in the changes of the relative abundance of *Firmicutes* at genus level between the kefir and unfermented milk groups (*p* > 0.05, for each) (Figure 3). Among the phyla *Actinobacteria*, the relative abundance of *Bifidobacterium* was increased from 31% to 39% by the intervention of kefir, and from 23% to 32% by the intervention of unfermented milk. However, these changes were not found significant when compared between the groups (*p* > 0.05). Furthermore, *Bifidobacterium* species were detectable in only 50% of participants’ the fecal samples at the baseline, whereas they could be detected in 91.7% after the intervention of kefir (data not shown). On the contrary, *Verrucomicrobia* was obtained less frequently from baseline (75% of participants) to after the intervention (58.3% of participants) in the kefir group. 

The correlation between the change in gut microbiota composition and the change in physiological characteristics, including anthropometrical measurements, biochemical markers, or blood pressure were conducted to examine the potential associations. Correlations between the changes in anthropometrical measurements and fecal microbiota composition at phylum and subphylum level were summarized in Table 3. The body weight and BMI were positively correlated with the relative abundance of *Firmicutes* and *Proteobacteria.* However, they were negatively correlated with the relative abundance of *Clostridia* (*p* < 0.05, for each). The body fat mass was negatively correlated with the relative abundance of *Bacteroidetes* (*p* < 0.01), and positively correlated with the relative abundance of *Porphyromonadaceae, Firmicutes*, and *Actinobacteria* (*p* < 0.05, for each). The waist circumference was negatively correlated with the relative abundance of *Clostridia* and positively correlated with the relative abundance *Veillonellaceae* (*p* < 0.05, for each).

Table 4 summarizes the correlation between the change in gut microbiota composition and biochemical markers. The changes in the relative abundance of *Bacteroides* was negatively correlated with both total and LDL cholesterol while the changes in the relative abundance of *Veillonellaceae* was negatively correlated with only LDL cholesterol (*p* < 0.05, for each). The change in the relative abundance of *Bacteroidetes* was negatively, and the change in the relative abundance of *Odoribacteraceae* and *Alistipes* groups was positively correlated with the change in serum glucose. The change in the relative abundance of *Verrucomicrobia* was positively correlated with changes in serum homocysteine and insulin (*p* < 0.05, for each). When we analyzed the correlation between the change in gut microbiota composition and blood pressure in the phylum level, the change in the relative abundance of *Actinobacteria* and *Proteobacteria* was positive, whereas *Bacteroidetes* was negatively correlated with the change in blood pressure. In the sub–phylum level, the change in the relative abundance *Lactobacillales* was positively correlated with the change in systolic and diastolic blood pressure (*p* < 0.05).

**Figure 4:**
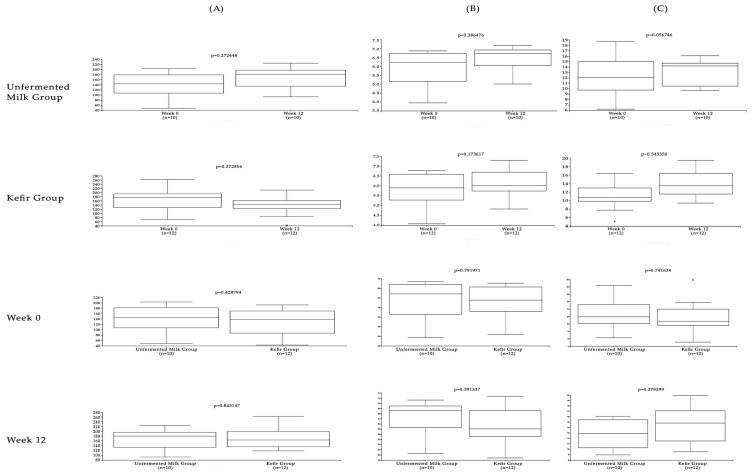
Box–and–whisker plots of the alpha diversity metrics calculated for the study groups. The box represents the median and the interquartile range, whereas whiskers indicate the 90th and 10th percentiles (**A**: Observed OTUs, **B**: Shannon index, **C**: Faith phylogenetic diversity index).

**Figure 5:**
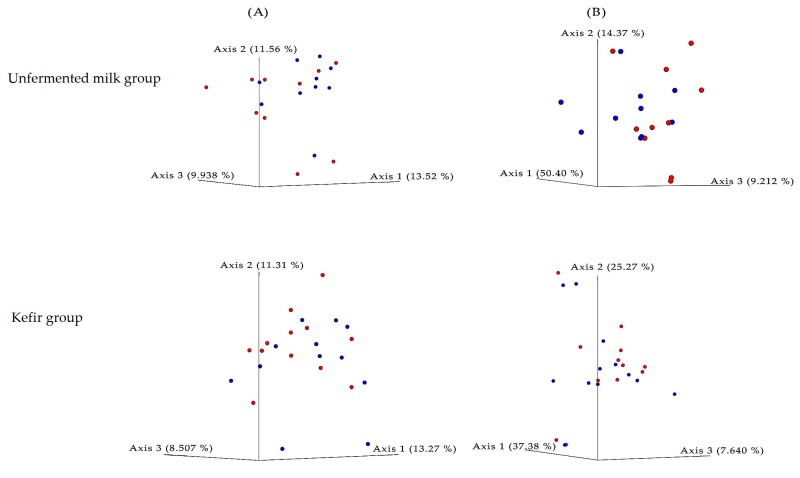
Principal coordinate analysis (PCoA) plot of the unweighted (**A**) and weighted (**B**) UniFrac distance matrices in the study groups. The plots were generated using EMPeror [36]. Axis titles indicate the percentage variations. The colors indicate sampling time (red: week 0, blue: week 12).

We further assessed several alpha and beta diversity metrics to assess bacterial biodiversity in specimens collected in week 0 and 12 from kefir and unfermented milk groups. No statistically significant differences were observed in OTU counts (Figure 4). Alpha diversity indices, indicating species richness and evenness with/without phylogenetic relations; namely, Shannon, Jaccard, and Faith PD indices, were similar between the study groups (Figure 5) (Jaccard plots not provided). Biodiversity between study cohorts, assessed by Bray–Curtis and weighted/unweighted UniFrac distances revealed no significant variation among study groups or in different time points. The PCoA plots of the unweighted UniFrac distances were given in Figure 5. No differences in OTU counts, alpha or beta diversity measures were observed when specimens from week 0 and 12 were assessed, regardless of the study group.

No side effect was reported by the participants during or after the intervention period that included the consumption of kefir.

## 4. Discussion

In this parallel–group randomized controlled study, regular kefir consumption during 12 weeks provided some improvements in anthropometrical measurements, lipid profile, glycaemic status, and inflammation in participants with MetS. In particular, insulin and HOMA–IR levels were significantly decreased, and also pro–inflammatory cytokines (TNF–α and IFN–γ) and blood pressure were ameliorated by kefir consumption. However, the magnitude of the improvements stayed insignificant when compared to unfermented milk. The effects of kefir on metabolic status were previously investigated in both animal models and human studies [37,38,39]. Some animal models suggested that kefir might have a potential to benefit the management of MetS by reducing body weight, fasting blood glucose, insulin, total, and LDL cholesterol, triacylglycerol, and pro–inflammatory cytokines, including IL–1β and IL–6 [38]. However, the evidence from human studies has been controversial. For instance, Ostadrahimi et al. reported that consumption of 600 mL/d kefir containing *Lactobacillus casei*, *Lactobacillus acidophilus*, and *Bifidobacteria* species had beneficial effects on fasting blood glucose and HbA1c compared to the control drink in patients with type 2 diabetes [37]. On the other hand, St–Onge et al. showed that 500 mL/day of kefir consumption for four weeks had no effect on lipid profile [40]. Furthermore, Fathi et al. showed that two servings of kefir in a day during eight weeks led a similar improvement both in lipid profile and weight management compared with milk [41,42]. The variation in response to the kefir consumption could be mainly explained by the variation of kefir composition, and the characteristics of study samples in different studies. Many different bacteria and yeast might be used for kefir production, and this might lead to distinct effects on metabolism and gut microbiota. Kefir used in this study contained *Lactococcus lactis* subsp. *lactis, Lactococcus lactis* subsp. *cremoris, Lactococcus lactis* subsp. *diacetylactis, Leuconostoc mesenteroides* subsp. *cremoris, Lactobacillus kefir, Kluyveromyces marxianus*, and *Saccharomyces unisporus,* and differed from the kefir samples used in other studies [37,38]. Furthermore, the initial metabolic profile of the participants was suggested an essential factor for the efficacy of probiotic interventions. Fuentes et al. showed that probiotics are more effective in patients with high baseline total cholesterol levels (251–300 mg/dL) compared to the patients with low baseline total cholesterol levels (200–250 mg/dL) [43]. Similarly, Nikbakht found out that probiotic supplementation was only effective in patients with baseline fasting blood glucose level above 126 mg/dL [44]. In our study, the median of total cholesterol levels was 243.50 mg/dL and 220.00 mg/dL, and the baseline glucose levels were 105.00 mg/dL and 101.50 mg/dL for kefir group and unfermented milk group, respectively. This may partly explain the lack of efficacy of kefir on metabolic status in our study. 

Alterations in gut microbiota diversity, composition, and function were suggested to play a significant role in the development of MetS [12]. Ameliorating the intestinal dysbiosis with prebiotics and probiotics have gained considerable attention in recent years for the management of MetS [45]. However, the studies have yielded inconsistent results regarding the influence of probiotics on fecal microbial diversity and composition [46,47,48,49,50,51,52]. Furthermore, the effects of kefir as a probiotic on gut microbiota have been examined very limited and mainly with animal studies. Kim et al. revealed that three-week oral administration of kefir provided a decrease in the number of *Firmicutes*, *Proteobacteria,* and *Enterobacteriaceae*, and an increase in the number of *Bacteroidetes*, *Lactobacillus*, *Lactococcus,* and total yeast compared to milk group in mice [53]. However, in their follow–up study, no significant difference apart from the increase in *Lactobacillus/Lactococcus* populations was observed in the kefir group compared to control [39]. Similarly, an increase in *Lactobacillus* and *Bifidobacterium* populations and a reduction in *Clostridium* populations by consumption of kefir have been reported in mice previously [54,55,56]. The present study is one of the first reports showing the impact of kefir on human microbiota composition in patients with MetS. In this study, regular kefir and milk consumption for 12 weeks resulted in some alterations in the gut microbiota composition. For instance, *Lactobacillus* and *Bifidobacterium spp.* were increased by kefir consumption. However, apart from the increase in the relative abundance of *Actinobacteria*, no significant change by kefir consumption was recorded. Furthermore, the changes in the relative abundance of bacterial populations did not differ significantly between the groups. In some studies, following the probiotic supplementation, increases in the supplemented genera without an additional impact on the main microbial groups were observed [57,58]. Previously, Yılmaz et al. showed that 400 mL/day kefir consumption for four weeks in patients with inflammatory bowel diseases resulted in the significant increase of Lactobacillus bacterial load in feces [59]. Our study also showed an increase from 2% to 5% in the relative abundance of *Lactobacillales* by kefir consumption, albeit lacking statistical significance. It has suggested that the change of the microbiota composition may be related to several factors such as age, gender, initial microbiota composition, dietary intake, lifestyle factors, menopausal status, and medical therapy of the individuals [60,61]. Moreover, the microbiota composition of the product (kefir) that was tested [17,18] and the consumption pattern, including the period and frequency of consumption and amount of the product should be considered as the factors that have potential to influence the magnitude of the changes in gut microbiota [62]. Therefore, evaluating the effect of probiotics or fermented foods such as kefir on an individual basis may be set as a goal for future studies. 

In this study, the correlations between changes in microbiota and anthropometric measurements or biochemical status were demonstrated. Our results pointed out a negative correlation between body fat mass and abundance of *Bacteroidetes*, whereas a positive correlation with the abundance of *Firmicutes* and *Actinobacteria* were observed. Although the data regarding the abundance of *Bacteriodetes* and *Firmicutes* phyla in obese and lean individuals is inconsistent, an overall analysis of results indicates an increase in *Firmicutes* with obesity [63]. Our results supported the previous reports, which revealed increased *Firmicutes* and decreased the abundance of *Bacteroidetes* are associated with obesity [64]. Turnbaugh et al. revealed a higher proportion of *Actinobacteria* in obese individuals compared to lean individuals [65]. In this study, we observed a positive correlation between body fat mass and abundance of *Actinobacteria,* which is in line with Turnbaugh et al.’s work. Members of the phylum of *Proteobacteria* are gram–negative bacteria and include several common human pathogens. An association between the increased relative abundance of *Proteobacteria* and increased risk of cardio–metabolic disorders was suggested previously [66]. In parallel with these findings, our study showed a positive correlation between an increased relative abundance of Proteobacteria and both body weight and diastolic blood pressure. 

In terms of glycaemic status, Larsen et al. showed a lower abundance of *Firmicutes* and *Clostridia,* and a higher abundance of *Bacteroidetes* and *Betaproteobacteria* in diabetic patients compared to the non–diabetics [67]. Accordingly, we observed a lower abundance of *Firmicutes* and a higher abundance of *Bacteroidetes* in both groups at the beginning of the study. However, only the change in the relative abundance of *Bacteroidetes* by the dietary intervention was negatively correlated with the change in fasting blood glucose. This was parallel to results of the study conducted by Egshatyan et al., which found that microbiota of glucose–intolerant subjects were represented by *Firmicutes* phylum and to a lesser degree by *Bacteroidetes* phylum [68]. In the subphylum level, studies mostly indicate higher levels of *Bacteroides* and *Prevotella* and lower levels of butyrate producing–bacteria in type 2 diabetic patients [68,69]. In this study, we have observed a positive correlation between the relative abundance of *Odoribacteraceae* and *Alistipes,* and fasting plasma glucose. The correlation between *Alistipes* and blood glucose was also observed in a previous study [70]. 

Many researchers demonstrated a link between dysbiosis of gut microbiota and blood pressure. Yang et al. reported an increase in *Firmicutes/Bacteroidetes* ratio in hypertensive rats and humans. They also recorded a lower abundance of *Actinobacteria* as well as acetate– and butyrate–producing bacteria [71]. Yan et al. indicated higher levels of *Proteobacteria* but lower levels of *Actinobacteria* in hypertensive subjects [72]. In this study, the change in the relative abundance of *Bacteroidetes* was negatively correlated with the change in systolic and diastolic blood pressure as reported previously. Surprisingly, we observed a strong positive correlation with systolic and a weak positive correlation with diastolic blood pressure, respectively, with *Actinobacteria* abundance. The phylum *Actinobacteria* includes *Bifidobacterium* genera, possessing probiotic features [73]. Studies that report reductions of *Actinobacteria* in hypertensive patients explained this relationship mostly with *Bifidobacterium* levels [71,74]. In our study, when we analyzed the association between *Bifidobacterium* and blood pressure, no significant correlation was noted. This may be due to the lack of significant changes in *Bifidobacterium* abundance after the intervention. The species other than *Bifidobacterium* within the *Actinobacteria* phylum might be further investigated in terms of their contributions to hypertension. 

Apart from fermentation, unfermented dairy products may also affect the gut microbiota [75,76]. In our study, regular milk consumption that was used as control also led to some changes in microbiota composition compared to the baseline. *Firmucutes* and *Verrucomicrobiota* were increased with milk consumption. However, *Bacteroidetes* group was decreased compared to the baseline. In accordance with our results, Ntemiri et al. also found out that whole milk consumption was associated with an increase in taxons belonging to *Firmicutes* and a higher *Firmicutes/Bacteroidetes* ratio [76]. In another randomized cross–over study, consumption of probiotic yogurt and milk acidified with D-(+)-glucono-δ-lactone showed some distinct effects on microbiota composition. In both groups, the abundance of *Bilophila wadsworthia* was reduced. However, only the abundance of *Bifidobacterium* species was increased with acidified milk intake, and it was suggested that gluconic acid in milk might possess prebiotic activity [75]. In a like manner, exopolysaccharides such as kefiran derived from kefir also suggested as bioactive compounds due to their potential prebiotic effects and relation to alteration of intestinal microbiota [77]. These results support a strong interaction between diet and microbiota even without probiotic intervention.

Studies showed that not only the composition of microbiota but also its functionality plays a role in the metabolic status [78,79]. This study focused only on the composition of gut microbiota; any consideration of its functionality was not taken into account. Using the metabolites of microbiota, such as postbiotics, as markers of the efficiency might have provided a better understanding. This should be noted as the main limitation of the study. The small number of participants in each arm could also be considered as the other limitation of the study.

## 5. Conclusions

In conclusion, to our knowledge, this was the first report exploring the effect of kefir on microbiota composition in patients with metabolic syndrome. This study indicated that kefir consumption could provide some potential improvements, especially in glycaemic status, inflammation–related indicators, and blood pressure, however, none of these improvements might stay significant when compared the changes led by unfermented milk consumption. Regarding to microbiota composition, the relative abundance of *Actinobacteria* phylum were increased in the kefir group compared to the baseline, even though a similar change by unfermented milk was also reported. Furthermore, this study underlined the potential alterations in gut microbiota composition that can be correlated with some indicators of the metabolic status led by both kefir and milk consumption, even if the magnitude of the efficiency remained limited. Further studies, especially randomized controlled trials, are needed to clarify the efficiency of kefir on gut microbiota and its link to metabolic status.

## Figures and Tables

**Table 1:** Baseline characteristics of kefir and unfermented milk groups.

Characteristics	Kefir Group	Unfermented Milk Group	
	Baseline	Baseline	*p*
**Sex (Female/Male)**	10/2	6/4	0.348
**Age (year)**	52.00 (47.50–60.50)	53.00 (45.00–60.00)	0.821
**Dietary intake**			
Energy (kcal/day)	1694.16 (1590.92–1936.72)	1655.35 (1423.52–2026.12)	0.821
Carbohydrate (g)	182.87 (166.79–205.74)	155.79 (141.61–224.90)	0.283
Protein (g)	73.12 (59.29–83.91)	65.23 (47.55–75.36)	0.254
Fat (g)	73.89 (68.16–97.67)	85.21 (68.06–105.53)	0.418
Fibre (g)	26.11 (18.42–36.90)	23.28 (17.11–26.23)	0.418
**Anthropometrical measurements**			
Weight (kg)	84.05 (69.23–88.78)	87.65 (75.60–100.60)	0.180
Body mass index (kg/m^2^)	30.67 (26.94–34.66)	32.38 (29.18–34.59)	0.381
Body fat mass (%)	37.05 (31.33–44.05)	37.45 (27.05–41.45)	0.582
Waist circumference (cm)	100.50 (90.75–110.00)	106.75 (102.25–119.00)	0.228
Hip circumference (cm)	111.50 (106.00–116.50)	112.00 (106.00–119.25)	0.771
Waist–to–hip ratio	0.92 (0.86–0.99)	0.97 (0.92–1.00)	0.203
**Lipid profile**			
Total cholesterol (mg/dL)	243.50 (217.25–265.25)	220.00 (199.75–249.00)	0.228
HDL cholesterol (mg/dL)	45.00 (39.00–55.75)	42.50 (34.50–56.25)	0.456
LDL cholesterol (mg/dL)	154.50 (135.75–177.00)	141.00 (114.50–177.50)	0.283
Triglycerides (mg/dL)	185.00 (114.50–216.75)	164.50 (126.25–220.75)	0.923
Homocysteine (µmoL/L)	10.01 (8.64–12.40)	13.10 (10.73–15.25)	0.050
**Glycaemic status**			
Glucose (mg/dL)	105.00 (93.75–109.75)	101.50 (97.00–107.25)	>0.99
Insulin (mU/L)	15.94 (11.75–17.64)	19.04 (18.09–25.49)	0.011 *
HbA1c (%)	5.60 (5.25–5.88)	5.65 (5.20–6.03)	0.872
HOMA–IR	4.18 (2.86–4.59)	4.52 (4.29–6.65)	0.180
**Inflammation–related indicators**			
hs–CRP (mg/dL)	0.22 (0.69–0.80)	0.27 (0.21–0.41)	0.722
TNF–α (pg/mL)	12.01 (0.76–43.05)	8.51 (0.49–25.85)	0.418
IL–6 (pg/mL)	15.82 (11.52–29.75)	19.73 (13.85–28.71)	0.418
IL–10 (pg/mL)	4.38 (1.13–32.90)	1.45 (1.13–9.34)	0.456
IFN–γ (IU/mL)	1.23 (0.12–2.19)	0.56 (0.02–3.04)	>0.99
ALT (U/L)	18.50 (16.50–24.00)	25.00 (20.75–31.25)	0.140
AST (U/L)	19.00 (18.00–20.00)	19.00 (18.00–20.25)	0.923
GGT (U/L)	15.00 (10.75–23.00)	19.00 (16.00–40.50)	0.169
**Blood pressure**			
Systolic blood pressure (mmHg)	134.50 (115.25–140.50)	132.50 (123.75–144.00)	0.722
Diastolic blood pressure (mmHg)	85.00 (77.50–92.00)	89.00 (81.00–92.00)	0.497

Data are given as median (25th percentile–75th percentile). Mann–Whitney U test was used to compare differences between groups. Fisher’s exact test was used to compare gender between groups. HDL: High Density Lipoprotein, LDL: Low Density Lipoprotein, HbA1c: Glycosylated Hemoglobin, HOMA-IR: Homeostasis Model of Assessment Insulin Resistance, hs-CRP: High-sensitivity C-reactive Protein, TNF: Tumor Necrosis Factor, IL: Interleukin, IFN: Interferon, ALT: Alanine Aminotransferase, AST: Aspartate Aminotransferase, GGT: Gamma-glutamyl Transferase. * *p* < 0.05.

**Table 2:** Dietary intake, anthropometrical measurements, biochemical parameters, and blood pressure in kefir and unfermented milk groups.

Characteristics	Kefir Group			Unfermented Milk Group			
	Baseline	Week 12	p^1^	Baseline	Week 12	p^2^	p^3^
**Dietary intake**							
Energy (kcal/day)	1694.16 (1590.92–1936.72)	1995.73 (1567.23–2351.80)	0.347	1655.35 (1423.52–2026.12)	1979.09 (1606.15–2123.62)	0.575	0.821
Carbohydrate (%)	44.00 (38.00–45.75)	42.50 (37.75–48.75)	0.964	41.00 (32.00–45.25)	46.00 (38.50–52.50)	0.214	0.283
Protein (%)	16.50 (15.25–19.00)	13.50 (12.00–18.50)	0.066	15.50 (12.75–17.25)	15.00 (12.00–16.50)	0.717	0.254
Fat (%)	39.50 (37.00–44.75)	41.00 (38.00–46.00)	0.666	43.50 (41.75–48.00)	40.00 (32.75–44.25)	0.167	0.228
Fibre (g)	26.11 (18.42–36.90)	26.81 (21.58–32.65)	0.814	23.28 (17.11–26.23)	25.17 (16.67–33.61)	0.646	0.722
**Anthropometrical measurements**							
Weight (kg)	84.05 (69.23–88.78)	83.50 (66.90–88.75)	0.695	87.65 (75.60–100.60)	88.55 (74.33–96.65)	0.207	0.418
Body mass index (kg/m^2^)	30.67 (26.94–34.66)	30.58 (26.24–34.31)	0.754	32.38 (29.18–34.59)	31.90 (29.05–33.71)	0.241	0.418
Body fat mass (%)	37.05 (31.33–44.05)	35.85 (30.58–44.23)	0.248	37.45 (27.05–41.45)	38.30 (29.63–43.98)	0.241	0.069
Waist circumference (cm)	100.50 (90.75–110.00)	102.25 (90.00–109.00)	0.407	106.75 (102.25–119.00)	106.75 (100.50–118.50)	0.952	0.722
Hip circumference (cm)	111.50 (106.00–116.50)	110.00 (106.25–118.63)	0.813	112.00 (106.00–119.25)	111.75 (105.13–116.25)	0.483	0.228
Waist–to–hip ratio	0.92 (0.86–0.99)	0.92 (0.86–0.95)	0.929	0.97 (0.92–1.00)	0.99 (0.92–1.03)	0.386	0.497
**Lipid profile**							
Total cholesterol (mg/dL)	243.50 (217.25–265.25)	222.00 (201.25–275.00)	0.209	220.00 (199.75–249.00)	226.50 (198.75–240.25)	0.953	0.539
HDL cholesterol (mg/dL)	45.00 (39.00–55.75)	46.00 (41.00–63.00)	0.271	42.50 (34.50–56.25)	43.50 (36.00–58.00)	0.412	0.346
LDL cholesterol (mg/dL)	154.50 (135.75–177.00)	144.00 (116.50–188.75)	0.170	141.00 (114.50–177.50)	147.50 (115.75–167.50)	0.959	0.314
Triglycerides (mg/dL)	185.00 (114.50–216.75)	152.50 (116.50–191.25)	0.530	164.50 (126.25–220.75)	161.50 (117.00–236.75)	0.878	1.000
Homocysteine (µmol/L)	10.01 (8.64–12.40)	9.31 (7.45–12.70)	0.182	13.10 (10.73–15.25)	12.00 (11.05–14.35)	0.213	0.710
**Glycaemic status**							
Glucose (mg/dL)	105.00 (93.75–109.75)	100.50 (96.50–103.00)	0.157	101.50 (97.00–107.25)	98.50 (97.50–116.25)	0.918	0.159
Insulin (mU/L)	15.94 (11.75–17.64)	13.64 (7.33–16.36)	0.050 *	19.04 (18.09–25.49)	22.08 (15.05–28.54)	0.386	0.123
HbA1c (%)	5.60 (5.25–5.88)	5.65 (5.50–5.98)	0.157	5.65 (5.20–6.03)	5.70 (5.10–5.90)	0.918	0.123
HOMA–IR	4.18 (2.86–4.59)	3.42 (1.93–4.22)	0.050 *	4.52 (4.29–6.65)	5.52 (3.38–8.49)	0.445	0.159
**Inflammation–related indicators**							
hs–CRP (mg/dL)	0.22 (0.69–0.80)	0.16 (0.10–0.46)	0.533	0.27 (0.21–0.41)	0.24 (0.13–0.50)	0.917	0.733
TNF–α (pg/mL)	12.01 (0.76–43.05)	1.13 (0.49–8.33)	0.015 *	8.51 (0.49–25.85)	4.12 (0.49–13.03)	0.401	0.123
IL–6 (pg/mL)	15.82 (11.52–29.75)	13.47 (5.65–21.39)	0.099	19.73 (13.85–28.71)	10.03 (6.16–16.45)	0.047 *	0.872
IL–10 (pg/mL)	4.38 (1.13–32.90)	1.91 (1.13–14.77)	0.386	1.45 (1.13–9.34)	1.13 (1.13–15.95)	0.735	0.539
IFN–γ (IU/mL)	1.23 (0.12–2.19)	0.38 (0.04–0.85)	0.013 *	0.56 (0.02–3.04)	0.49 (0.18–1.19)	0.086	0.628
ALT (U/L)	18.50 (16.50–24.00)	22.00 (19.50–24.00)	0.288	25.00 (20.75–31.25)	24.50 (18.75–29.00)	0.215	0.180
AST (U/L)	19.00 (18.00–20.00)	19.00 (17.00–22.50)	0.887	19.00 (18.00–20.25)	17.50 (17.00–19.50)	0.136	0.203
GGT (U/L)	15.00 (10.75–23.00)	14.50 (12.00–23.75)	0.371	19.00 (16.00–40.50)	19.00 (14.25–29.25)	0.065	0.169
**Blood pressure**							
Systolic blood pressure (mmHg)	134.50 (115.25–140.50)	118.00 (103.25–137.75)	0.041 *	132.50 (123.75–144.00)	118.00 (105.75–137.00)	0.047 *	0.974
Diastolic blood pressure (mmHg)	85.00 (77.50–92.00)	78.50 (69.00–80.00)	0.019 *	89.00 (81.00–92.00)	78.50 (66.75–89.50)	0.059	1.000

Data are given as median (25^th^ percentile–75^th^ percentile). HDL: High Density Lipoprotein, LDL: Low Density Lipoprotein, HbA1c: Glycosylated Hemoglobin, HOMA-IR: Homeostasis Model of Assessment Insulin Resistance, hs-CRP: High-sensitivity C-reactive Protein, TNF: Tumor Necrosis Factor, IL: Interleukin, IFN-γ: Interferon-γ, ALT: Alanine Aminotransferase, AST: Aspartate Aminotransferase, GGT: Gamma-glutamyl Transferase. p^1^ gives the differences between baseline and after intervention in the kefir group. p^2^ gives the differences between baseline and after intervention in the unfermented group. p^3^ gives the comparison of the changes from baseline to week 12^th^ between groups. p^1^ and p^2^ were analysed by the Wilcoxon test while p^3^ was analyzed by the Mann–Whitney U test. * *p* ≤ 0.05.

**Table 3:** Correlations between changes in anthropometrical measurements and fecal microbiota composition at phylum and subphylum level ^≠^.

	Bacteroidetes	Bacteroides	Odoribacteraceae	Porphyromonadaceae	Prevotellaceae	Alistipes	Firmicutes	Clostridia	Erysipelotrichaceae	Veillonellaceae	Lactobacillales	Verrucomicrobia	Actinobacteria	Bifidobacterium	Proteobacteria
**Anthropometric measurements**															
Weight (kg)	−0.384	0.070	0.176	0.160	−0.131	0.133	0.433 *	−0.432 *	−0.338	0.295	0.352	0.132	0.400	0.030	0.456 *
Body mass index (kg/m^2^)	−0.383	0.103	0.189	0.172	−0.168	0.139	0.434 *	−0.455 *	−0.341	0.291	0.347	0.095	0.382	0.047	0.461 *
Body fat mass (%)	−0.563 **	0.223	0.285	0.468 *	−0.388	0.402	0.599 **	0.017	−0.070	0.210	0.038	0.420	0.536 *	−0.018	0.371
Waist circumference (cm)	−0.151	0.267	−0.321	−0.009	−0.217	−0.047	0.242	−0.505 *	−0.287	0.432 *	0.135	0.247	0.128	0.044	0.332
Hip circumference (cm)	0.082	0.029	0.326	0.164	−0.149	−0.078	0.070	−0.096	−0.264	0.272	0.215	−0.115	0.127	−0.367	0.081
Waist–to–hip ratio	−0.239	0.112	−0.526 *	−0.086	−0.038	0.014	0.168	−0.251	0.046	0.066	−0.043	0.376	0.090	0.223	0.208

**^≠^** Values indicate Spearman correlation coefficients (*n* = 22). * Correlation is significant at the 0.05 level (2–tailed). ** Correlation is significant at the 0.01 level (2–tailed).

**Table 4:** Correlations between changes in biochemical markers and fecal microbiota composition at phylum and sub–phylum level ^≠^.

	Bacteroidetes	Bacteroides	Odoribacteraceae	Porphyromonadaceae	Prevotellaceae	Alistipes	Firmicutes	Clostridia	Erysipelotrichaceae	Veillonellaceae	Lactobacillales	Verrucomicrobia	Actinobacteria	Bifidobacterium	Proteobacteria
**Lipid profile**															
Total cholesterol (mg/dL)	−0.151	−0.444 *	−0.025	−0.026	0.334	−0.007	−0.101	0.389	0.205	−0.408	0.009	0.059	0.190	−0.164	−0.096
HDL cholesterol (mg/dL)	−0.003	−0.418	0.130	−0.066	0.414	−0.004	−0.248	−0.224	0.161	0.154	0.259	0.175	−0.016	−0.012	0.130
LDL cholesterol (mg/dL)	−0.016	−0.535 *	−0.021	−0.023	0.305	0.075	−0.298	0.322	0.330	−0.431 *	0.005	0.152	0.042	−0.034	−0.094
Triglycerides (mg/dL)	−0.080	−0.007	−0.118	−0.079	0.197	−0.263	0.251	0.334	−0.211	−0.242	−0.082	−0.176	0.199	−0.292	−0.210
Homocysteine (µmoL/L)	−0.162	0.077	−0.186	0.141	−0.135	−0.023	−0.057	−0.044	0.232	0.224	0.149	0.477 *	0.110	0.127	0.359
**Glycaemic status**															
Glucose (mg/dL)	−0.590 **	0.092	0.423 *	0.370	−0.374	0.629 **	0.387	0.317	0.056	−0.037	−0.033	0.328	0.365	0.090	0.187
Insulin (mU/L)	−0.331	0.069	0.344	0.244	−0.265	0.361	0.357	−0.098	−0.159	0.312	0.010	0.428 *	0.284	−0.218	0.123
HbA1c (%)	0.189	0.062	−0.168	−0.266	0.004	−0.123	0.021	0.126	−0.372	0.211	−0.282	−0.003	−0.280	0.068	−0.277
HOMA–IR	−0.356	0.030	0.406	0.207	−0.255	0.458 *	0.346	−0.119	−0.148	0.269	0.067	0.395	0.294	−0.209	0.123
**Inflammation Related Indicators**															
hs–CRP (mg/dL)	−0.145	0.110	−0.294	0.238	−0.255	−0.032	0.096	0.145	0.358	0.078	−0.015	0.380	0.321	−0.289	0.078
TNF–α (pg/mL)	−0.297	0.087	−0.305	−0.034	−0.224	0.051	0.010	0.317	0.093	0.023	−0.062	0.392	0.281	−0.323	0.322
IL–6 (pg/mL)	−0.016	0.381	0.001	0.013	−0.287	−0.142	0.313	−0.320	−0.333	0.399	−0.170	0.039	−0.043	0.091	0.274
IL–10 (pg/mL)	0.086	−0.130	−0.414	−0.314	0.165	−0.216	−0.054	−0.289	−0.040	0.015	0.273	0.151	−0.006	0.008	−0.054
IFN–γ (IU/mL)	0.066	−0.241	−0.089	−0.074	0.111	0.291	−0.182	−0.024	0.074	−0.162	0.084	0.093	−0.029	0.086	−0.076
AST	−0.343	0.292	0.016	0.058	−0.159	−0.178	0.283	−0.160	−0.031	−0.006	0.203	−0.328	0.190	−0.187	0.169
ALT	−0.251	0.235	−0.089	0.067	−0.081	−0.423 *	0.391	0.138	−0.120	0.253	0.098	0.158	0.252	−0.402	0.175
GGT	0.255	−0.264	0.112	−0.099	0.493 *	−0.385	−0.093	0.025	−0.285	−0.089	−0.093	−0.457 *	−0.016	−0.169	−0.208
**Blood Pressure**															
Systolic blood pressure (mmHg)	−0.531 *	−0.148	−0.080	0.137	0.092	0.076	0.243	−0.172	0.144	−0.198	0.536 *	0.205	0.710 **	−0.168	0.379
Diastolic blood pressure (mmHg)	−0.491 *	0.169	0.183	0.271	−0.348	0.171	0.244	−0.205	0.239	−0.128	0.561 **	0.096	0.452 *	0.013	0.469 *

**^≠^** Values indicate Spearman correlation coefficients (*n* = 22). * Correlation is significant at the 0.05 level (2–tailed). ** Correlation is significant at the 0.01 level (2–tailed). HDL: High Density Lipoprotein, LDL: Low Density Lipoprotein, HbA1c: Glycosylated Hemoglobin, HOMA-IR: Homeostasis Model of Assessment Insulin Resistance, hs-CRP: High-sensitivity C-reactive Protein, TNF: Tumor Necrosis Factor, IL: Interleukin, IFN: Interferon, ALT: Alanine Aminotransferase, AST: Aspartate Aminotransferase, GGT: Gamma-glutamyl Transferase.
